# Welcome Message From the New Editor‐in‐Chief of Digestive Endoscopy Open

**DOI:** 10.1002/deo2.70387

**Published:** 2026-07-30

**Authors:** Kazuki Sumiyama

**Affiliations:** ^1^ Department of Endoscopy The Jikei University School of Medicine Tokyo Japan

It is an extraordinary honor and privilege to be appointed as the Editor‐in‐Chief of DEN Open, the official open‐access journal of the Japan Gastroenterological Endoscopy Society (JGES). First and foremost, I would like to express my deepest respect and gratitude to my predecessor, Professor Takao Itoi, the inaugural Editor‐in‐Chief, for his outstanding leadership and vision in successfully launching and establishing this journal. I am also profoundly grateful to Professor Mitsuhiro Fujishiro, President of JGES; Professor Masayuki Kitano, Editor‐in‐Chief of our sister journal, Digestive Endoscopy (DEN); and Professor Takayuki Matsumoto, the former Editor‐in‐Chief of DEN and the advisor of the journal, for providing me with this incredible opportunity to lead the journal into its next chapter.

## “Big Things Have Small Beginnings.”

Despite its short history since its launch in April 2021, DEN Open has already published a wealth of high‐quality original articles, clinically informative reviews, insightful editorials, and valuable case series and reports. As an endoscopist, I have witnessed firsthand how rapidly our field evolves and how diverse clinical insights, preserved in digital archives, shape and improve our day‐to‐day practice. We have been emphasizing the importance of case report contributions that are too often underestimated in publishing nowadays, because we know that documenting rare clinical scenarios could directly help patients suffering from unusual conditions now and in the future. I strongly encourage authors, especially our younger colleagues from around the world, to submit their work and unique clinical experiences to DEN Open, regardless of the study size or levels of evidence. Every clinical case holds the potential to teach us something valuable. We firmly believe that small insights obtained from individual patients can pave the way for major advancements in patient care globally.

## Our Commitment to Authors

To support our contributing authors, the editorial board of DEN Open is dedicated to maintaining an exceptional standard of peer review while deeply respecting your time. Our esteemed board of reviewers and editors ensures that every submission is carefully evaluated and polished to maximize its quality, clarity, and ultimate impact upon publication. We are committed to a rapid decision‐making process and swift publication timelines without any unnecessary delays.

Together with our exceptional editorial team, dedicated reviewers, and the steadfast support of JGES, I am fully committed to fostering DEN Open as a dynamic, inclusive, and highly influential platform in our field.

We look forward to receiving your submissions and working together to advance the frontiers of gastroenterological endoscopy.

Kazuki Sumiyama, MD, PhD, FJGES, FASGE

Editor‐in‐Chief, DEN Open, Professor and Chair, Department of Endoscopy, The Jikei University School of Medicine.

## Author Contributions

**Kazuki Sumiyama**: writing – original draft.

## Funding

The author has nothing to report.

